# Prevalence of the metabolic syndrome among children from six cities of China

**DOI:** 10.1186/1471-2458-12-13

**Published:** 2012-01-06

**Authors:** Haiquan Xu, Yanping Li, Ailing Liu, Qian Zhang, Xiaoqi Hu, Hongyun Fang, Tingyu Li, Hongwei Guo, Ying Li, Guifa Xu, Jun Ma, Lin Du, Guansheng Ma

**Affiliations:** 1 National Institute for Nutrition and Food Safety, Chinese Center for Disease Control and Prevention, 29 Nan Wei Road, Beijing, China; 2 Chongqing Children's Hospital, 136 Zhong Shan Er Road, Chongqing, China; 3 School of Public Health, Fudan University, 138 Yi Xue Yuan Lu, Shanghai, China; 4 Haerbin Medical University, 157 Bao Jian Road, Haerbin, China; 5 Department of Public Health, Shandong University, 44 Wen Hua Xi Lu, Jinan, China; 6 Institute of Children and Adolescent Health, Peking University Health Science Center, 38 Xue Yuan Road, Beijing, China; 7 Guangzhou Center for Disease Control and Prevention, 23 Zhong Shan San Lu, Guangzhou, China; 8 National Institute for Nutrition and Food Safety, Chinese Center for Disease Control and Prevention, 7 Pan Jia Yuan Nan Li, Beijing 100021, China

## Abstract

**Background:**

Metabolic syndrome (MetS) in childhood can increase the risk of cardiovascular disease, diabetes mellitus and dyslipidemia in adulthood, which is of increasing concern in transitional and advanced economies. The aim of the current study was to explore the prevalence of MetS among children from six cities of China.

**Methods:**

A total of 8,764 children (4,495 boys, 4,269 girls) aged 7-11 years were randomly selected from 6 cities of China. MetS was mainly defined by the criteria proposed by International Diabetes Federation (IDF).

**Results:**

The overall prevalence of MetS for children older than 10 years was 0.8% by IDF definition. Obese children had significantly higher MetS prevalence compared with their counterparts with overweight (6.6% vs. 0.9%, *p *< 0.01) and normal weight (6.6% vs. 0.05%, *p *< 0.01). The prevalence of abdominal obesity, high triglycerides, low high density lipoprotein cholesterol, hypertension and high glucose among obese children was 93.4%, 16.5%, 14.3%, 7.3% and 4.0%, respectively, which significantly higher than those among overweight children (37.0%, 6.1%, 10.0%, 4.2%, and 3.3%, respectively) and among normal weight children (1.2%, 3.3%, 4.0%, 1.7% and 2.5%, respectively). The proportion of children with at least one, two, and three items of the metabolic abnormalities were 25.0%, 5.4% and 0.9%, respectively. Metabolic abnormalities were also present in children under 10 years of age.

**Conclusions:**

The early onset of MetS among children and relatively high proportions of children with at least one or two metabolic abnormalities in cities of China can increase the risk of developing MetS. It implies the necessity to take effective actions to control and prevent the rapid development of obesity among children in developing countries, especial those undergoing transition to a Western lifestyle.

## Background

Metabolic syndrome (MetS) consists of abdominal obesity, hypertension, glucose intolerance and dyslipidemia (elevated triglycerides and decreased high-density lipoprotein cholesterol concentrations) [1]. It is a clustering of metabolic abnormalities associated with risk of coronary heart disease, stroke and type 2 diabetes [2-6]. Many studies have shown that along with the rising prevalence of obesity, MetS has been increasing as well [7]. Previous studies have revealed an association between obesity and the clustering of metabolic abnormalities in early life and their persistence during adulthood [8-15]. Children with MetS are at increased risk of developing type 2 diabetes and cardiovascular disease in later life [16,17].

In 2007, the International Diabetes Federation (IDF) released its guidelines for defining and diagnosing MetS in children and adolescents with the goal of developing a diagnostic tool for early detection of MetS [18]. IDF's definition includes waist circumference as a prerequisite, but otherwise adheres to the adult values for hypertension, glucose intolerance, and dyslipidemia. Many countries have reported the prevalence of MetS among children and adolescents. In China, the national data about MetS among adolescents can be found, however, the national data about MetS among young children are lacking. Therefore, the aim of the current study was to investigate the prevalence and distributions of MetS among children aged 7-11 years from six cities of China and provide information for decision makers for developing an effective intervention strategy and measurement.

## Methods

### Subjects' selection

Six provincial capital cities were selected in the study, i.e. Haerbin (Northeast China), Beijing (North China), Jinan (East China), Shanghai (East China), Chongqing (West China) and Guangzhou (South China). A stratified cluster nested sampling method was used for subject selection. From each city, 6 schools were selected randomly from all primary schools excluding special schools for disabled children. Two classes from each grade were selected randomly from grade 1-5 of each selected school. All students are free from physical disability, congenital diseases and any diagnosed medical condition that might potentially interfere with metabolism in the two classes were invited to participate in the study.

The total sample size was 9,866. Among them, 8,898 students (90.2%) gave both blood samples and physical measurements. There were no significant difference in the age, gender, height and weight between response participants and no-response participants. As the number of students in group aged 12 and 13 was too few (< 150) to be representative, they were excluded from the analysis. Finally, a total of 8,764 children (4,495 boys, 4,269 girls) aged 7-11 years were involved in the analysis (Table 1). The proportion of Han ethnicity was 97.0%, which was consistent with the composition of ethnic groups in China. The information on education and income level of the parents was collected from parents by using a self-administrated questionnaire. The information on the status of puberty was inquired by the trained interviewers. It took 2 months to finish the physical examination and questionnaire survey. The details of the protocol can be found somewhere else [19].

**Table 1:** The number of children with different ages

Age(years)	Boys	(pubertal)	Girls	(pubertal)	All	(pubertal)
7	1,018	(3)	1,011	(0)	2,029	(3)
8	1,082	(8)	1,015	(0)	2,097	(8)
9	1,001	(5)	990	(4)	1,991	(9)
10	919	(19)	843	(29)	1,762	(48)
11	475	(4)	410	(63)	885	(67)
All	4,495	(39)	4,269	(96)	8,764	(135)

The protocol of the study was approved by the Ethical Committee of the National Institute for Nutrition and Food Safety, Chinese Center for Disease Control and Prevention. Signed consent forms were obtained from both the children's parents or guardians and the children themselves.

### Anthropometric measurements

Fasting body weight was measured to the nearest 0.1 kg on a double ruler scale (RGT-140, Wujin Hengqi Co. Ltd., Changzhou, China). Height was measured to an accuracy of 1 mm with a freestanding stadiometer mounted on a rigid tripod (GMCS-I, Xindong Huateng Sports Equipment Co. Ltd., Beijing, China) by trained interviewers following a standardized procedure. BMI was calculated as weight in kilograms divided by height in meters squared (kg/m^2^).

Using age- and sex-specific BMI cutoff points developed by the Working Group for Obesity in China (WGOC), normal weight was defined as BMI for age and sex ≤ 85th percentile, overweight was defined as BMI between the 85th and the 95th percentiles, whereas obesity was defined as BMI ≥ 95th percentile [20].

Waist circumference (WC) was measured mid-way between the lower rib margin and the iliac crest with flexible anthropometric tape (Myotape). The WC was measured twice to the nearest 0.1 cm. If the variation between these two measurements was greater than 0.5 cm, a third measurement was taken and the mean was calculated by using the two closest measurements.

### Blood pressure

Blood pressure was measured in the seated position using a mercury sphygmomanometer by trained nurses with at least a 10-min rest period before the measurement. The first and the fifth Korotkoff sounds were used to represent the systolic and diastolic blood pressure. Three measurements were taken for all subjects at 2-min intervals, and the average of the last two measurements was used. The two nearest measurement (≤ 2 mmHg) were recorded, and their means were used for the analysis.

### Glucose and lipid profiles

Fasting venous blood samples (5 mL) were drawn in the morning after 10-14 h of overnight fasting. Serum glucose was determined by the glucose-oxidase method (Daiichi Pharmaceutical Co., Ltd, Tokyo, Japan) within 4 h after the sample was obtained. Total cholesterol (TC), triglycerides (TG), low-density lipoprotein cholesterol (LDL-C) and high-density lipoprotein cholesterol (HDL-C) were determined by enzymatic methods using commercial kits (Daiichi Pharmaceutical Co., Ltd, Tokyo, Japan).

### Quality control

About 20 technicians were involved in the survey in each city. The dedicated technicians were responsible for implementing each part of the survey. All the study group members from the collaboration centers had attended the training session and the study was completed following the same standardized procedure in each center. Then, the data was entered into computer using the EPIDATA software. The same equipments were provided and employed in the six study sites and all the measurements were taken following the standardized operating procedures by the trained technicians. The duplicate measurements in subgroups showed high reproducibility (correlation coefficients of duplicate measurements were 0.99 for height and 0.98 for weight). Every tenth sample was measured twice (correlation coefficient of duplicate measurements was 0.98).

### Definition of MetS

MetS was identified mainly according to the IDF definition [18] by abdominal obesity (≥ 90th percentile as assessed by WC), as determined by the cut-off points for Chinese children [21], and the presence of two or more clinical features including TG ≥ 1.7 mmol/L, HDL-C < 1.03 mmol/L, systolic blood pressure ≥ 130 mmHg and/or diastolic blood pressure ≥ 85 mmHg, and serum fasting glucose ≥ 5.6 mmol/L.

In order to be comparable with other publications, we also defined the MetS by other criteria, as proposed by Cook et al., de Ferranti et al. and Ohzeki T et al., respectively, in the sensitivity analysis [22-24].

### Statistical analysis

The prevalence of MetS was estimated by sex, age and BMI. Stratified analyses were performed with adjustment for age and sex. Prevalence values were compared using generalized linear mixed model (GLMM). Considering the complex study design, GLMM was used to compare the fixed effect after controlling for random effects. The fixed effects included sex, age, BMI, mother's education and family's income level. The class was taken as random effect. The means of continuous variables were compared using mixed linear model (MIXED). All statistical analyses were performed with SAS (9.2e for Windows; SAS Institute Inc. Cary, NC, USA), and the significance level was set at 0.05.

## Results

The characteristics of the subjects were shown in Table 1. Boys had a higher value in height, weight, WC, BMI, systolic blood pressure, HDL-C and serum glucose, and lower value in TG, TC and LDL-C as compared with their female counterparts (Table 2). A significant difference in socioeconomic level among the cities was found (*p *< 0.01). Among children less than 10 years old, 0.5% of boys and 0.1% of girls showed the first nocturnal emission or menarche. The proportions were 1.7% for boys and 7.4% for girls aged 10-11 years, respectively. The overall prevalence of overweight and obesity was 11.5% and 10.3%, respectively. Both overweight and obesity were significantly prevalent among boys than among girls (13.3% vs. 9.5%; 12.6% vs. 7.9%).

**Table 2:** Characteristics of the Subjects

	Boys (n = 4,495)	Girls (n = 4,269)	Total (n = 8,764)	
	Mean ± SD	Mean ± SD	Mean ± SD	*p*-value
Age (years)	8.7 ± 1.4	8.6 ± 1.4	8.6 ± 1.4	< 0.0001
Anthropometric measures				
Height (cm)	135.4 ± 9.4	134.6 ± 9.8	135.0 ± 9.6	< 0.0001
Weight (kg)	32.5 ± 9.6	30.4 ± 8.4	31.5 ± 9.1	< 0.0001
Waist circumference (cm)	59.3 ± 9.6	56.0 ± 7.7	57.7 ± 8.9	< 0.0001
BMI (kg/m^2^)	17.4 ± 3.3	16.5 ± 2.8	17.0 ± 3.1	< 0.0001
Blood pressure				
Systolic (mmHg)	100.9 ± 10.6	98.8 ± 10.6	99.9 ± 10.7	< 0.0001
Diastolic (mmHg)	63.6 ± 8.8	63.6 ± 9.2	63.6 ± 9.0	0.8478
Metabolic profile				
TG (mmol/L)	0.80 ± 0.46	0.83 ± 0.43	0.81 ± 0.45	< 0.0001
TC (mmol/L)	4.06 ± 0.76	4.11 ± 0.81	4.08 ± 0.78	0.0042
LDL-C (mmol/L)	2.08 ± 0.62	2.16 ± 0.66	2.12 ± 0.64	< 0.0001
HDL-C (mmol/L)	1.48 ± 0.31	1.45 ± 0.30	1.46 ± 0.31	< 0.0001
Glucose (mmol/L)	4.57 ± 0.57	4.45 ± 0.58	4.51 ± 0.58	< 0.0001
BMI status				
Normal weight (%)	74.1	82.6	78.2	< 0.0001
Overweight (%)	13.3	9.5	11.5	
Obesity (%)	12.6	7.9	10.3	

Comparison between boys and girls, using generalized linear mixed model for classified variables and mixed linear model for continuous variable by controlling the different schools and classes.

The overall prevalence of MetS was 0.8% among children aged 10-11 years and 0.5% among children aged 7-9 years according to the IDF definition (*p *< 0.05) (Table 3). The prevalence of MetS among children older than 10 years ranged from 0.3% to 1.5% in different cities (*p *> 0.05). No significant difference in MetS prevalence were found between boys and girls aged 10-11 years (*p *> 0.05). Obese children had significantly higher prevalence of MetS as compared to their counterparts with normal weight (*p *< 0.01). No significant associations were found between the MetS and the mother's education and family income level.

**Table 3:** The Prevalence of the Metabolic Syndrome among children

	N	MetS	Abdominal obesity	High TG level	Low HDL-C level	Elevated blood pressure	High glucose level
Children ≥ 10 years old							
Total	2,647	0.8	15.1	5.0	5.8	2.6	2.7
^a^Sex							
Boy	1,394	1.0	17.1	5.2	5.9	2.5	3.0
Girl	1,253	0.6	12.9	4.7	5.7	2.7	2.5
^ab^BMI status							
Normal weight	2,044	0.05**	1.2*	3.3**	4.0**	1.7**	2.5
Overweight	330	0.9	37	6.1	10	4.2	3.3
Obesity	273	6.6	93.4	16.5	14.3	7.3	4.0
^ab^Mother's educational level							
Low (illiterate)	28	3.6	17.9	14.3	7.1	0	0
Middle (Primary or junior middle school)	1,138	0.8	12.3	5.1	5.9	2.2	2.4
High (Senior middle school or above)	1,111	1.0	17.1	5.5	5.1	3.0	3.0
^ab^Family's economic level							
(Yuan/month/per family member)							
≤ 1,500	1,042	0.9	14.4	5.3	5.4	2.8	3.1
1,501-2,500	617	1.3	14.6	5.5	6.8	2.4	1.8
> 2,500	610	0.5	15.2	4.9	5.2	2.8	3.0
Children < 10 years old							
Total	6,117	0.5	13.0	3.5	5.0	1.5	1.9
^a^Sex							
Boy	3,101	0.5	13.6**	3.8	4.5	1.6	2.4**
Girl	3,016	0.4	12.2	3.2	5.5	1.3	1.4
^ab^BMI status							
Normal weight	4,813	0.04**	0.8**	2.6**	4.6**	0.7**	1.9
Overweight	675	0.4	29.9	3.7	4.1	3.1	1.6
Obesity	629	4.0	87.9	10.0	8.7	5.4	2.4
^ab^Mother's educational level							
Low (illiterate)	52	0	11.5	1.9	3.8	0	1.9
Middle (Primary or junior middle school)	1,854	0.8	12.2	4.1	5.4	1.5	1.8
High (Senior middle school or above)	2,276	0.5	15.9	3.2	4.7	1.8	2.2
^ab^Family's economic level							
(Yuan/month/per family member)							
≤ 1,500	1,808	0.5	14.7	3.8	5.3	1.3	1.6
1,501-2,500	1,145	0.7	13.9	3.1	4.2	1.5	2.3
> 2,500	1,171	0.7	14.3	3.5	5.6	2.2	2.6

*p < 0.05, **p < 0.01, compared using generalized linear mixed model

^a^Adjusted for age and puberty for comparison of different groups

^b^Adjusted for sex and puberty for comparison of different groups

^ab^Adjusted for age, sex and puberty for comparison of different groups

The prevalence of abdominal obesity, high triglycerides level, low HDL-C level, hypertension and high glucose level among children aged 10-11 years was 15.1%, 5.0%, 5.8%, 2.6% and 2.7%, respectively (Table 3). The prevalence of high triglycerides, low HDL-C levels, hypertension and high glucose among children with normal weight were all significant lower than that among overweight and obese children. Compared with children with normal weight, the odds ratio of having hypertension was 3.5 (95% confidence interval (CI): 2.3-5.4) for overweight children and 6.4 (95%CI: 4.4-9.3) for obese children after adjusting the age, sex and puberty. A significantly higher prevalence of abdominal obesity and abnormal glucose level were found among boys than among girls. Children older than 10 years had significantly higher prevalence of abdominal obesity, high triglycerides level and elevated blood pressure level as compared with their counterparts aged less than 10 years old. No significant associations were found between all MetS components and mother's education and family's income level.

The proportions of subjects with at least one, two and three metabolic abnormalities were shown in Table 4. Significantly higher proportions with at least one, two and three abnormalities were found among both the overweight and obese group as compared with their counterparts with normal weight. The proportion of boys with at least two metabolic abnormalities was significantly higher than that of girls. The proportions of older children with at least one, two and three metabolic abnormalities were significantly higher than that of their younger counterparts.

**Table 4:** Metabolic syndrome components among Chinese children (%)

	N	MetS components		
		≥ 1	≥ 2	≥ 3
Children ≥ 10 years old				
Total	2,647	25.0	5.4	0.9
^a^Sex				
Boy	1,394	26.1	6.7*	1.0
Girl	1,253	23.8	3.9	0.8
^ab^BMI status				
Normal weight	2,044	11.6**	0.9**	0.1**
Overweight	330	50.0	9.4	1.2
Obesity	273	95.2	33.7	6.6
^ab^Mother's educational level				
Low (illiterate)	28	28.6	7.1	3.6
Middle (Primary or Junior middle school)	1,138	22.0	4.9	1.0
High (Senior middle school or above)	1,111	26.6	6.0	1.0
^ab^Family's economic level				
(Yuan/Month/per family member)				
≤ 1,500	1,042	24.6	5.5	0.9
1,501-2,500	617	24.0	5.8	1.3
> 2,500	610	24.9	5.4	0.8
Children < 10 years old				
Total	6,117	20.7	3.5	0.5
^a^Sex				
Boy	3,101	21.5	3.9*	0.5
Girl	3,016	19.9	3.2	0.5
^ab^BMI status				
Normal weight	4,813	9.6**	0.9**	0.04**
Overweight	675	36.6	5.3	0.6
Obesity	629	88.7	21.8	4.0
^ab^Mother's educational level				
Low (illiterate)	52	19.2	0	0
Middle (Primary or Junior middle school)	1,854	20.2	4.0	0.8
High (Senior middle school or above)	2,276	22.8	4.4	0.5
^ab^Family's economic level				
(Yuan/Month/per family member)				
≤ 1,500	1,808	22.5	3.7	0.5
1,501-2,500	1,145	20.5	3.8	0.7
> 2,500	1,171	22.2	5.2	0.8

*p < 0.05, **p < 0.01, compared using generalized linear mixed model

^a^Adjusted for age and puberty for comparison of different groups

^b^Adjusted for sex and puberty for comparison of different groups

^ab^Adjusted for age, sex and puberty for comparison of different groups

In the sensitivity analysis, we presented the MetS defined by different criterion. The prevalence of MetS was 2.5%, 2.6% and 11.6% among children aged 10-11 years old according to criteria recommended by Cook et al., de Ferranti et al. and Ohzeki et al., respectively. (Additional File 1: Table S1)

## Discussion

To the best of our knowledge, the current study is the first study exploring the prevalence of MetS using the IDF criteria among children aged 7-11 years living in the urban areas of China. The prevalence of both MetS and its five individual components were much higher among overweight and obese children than that among normal weight children regardless of criteria.

Data about MetS in children and adolescents are limited. This is partly due to the lack of consensus on the definition of MetS for children. The prevalence varies depending on the criteria applied. The main criteria of MetS used in our study were proposed by the IDF in 2007, which had been used worldwide for comparison of data from different countries [25]. After IDF issued its MetS definition, the criteria were referenced by many researchers in different countries [25-29]. In 2009, Zorzi A et al. reported that the prevalence of MetS in Canadian Tsimshian Nation youth aged 6-18 years was 4.7% with the IDF criteria and it increased to 8.3% when pediatric hypertension norms were applied [27]. In 2009, Cizmecioglu et al. reported that the prevalence of MetS among 2,491 Turkish school children aged 10-19 years was 2.3% according to IDF's guidelines [29]. Compared with the above two studies, the prevalence of MetS among Chinese urban children was lower. This can be explained partially by the lower prevalence of obesity among Chinese children. The results from both our study and the previous studies indicated that obesity could increase the risk of developing MetS. In the current study, the prevalence of MetS among obese children was more serious than that among children with normal weight. This finding consisted with another study in Beijing in 2004 [30]. In 2010, Druet reported that the frequency of IDF-MetS was 8.9% in 300 overweight and obese French children aged 10-16 years old [26]. In 2010, another cross-sectional study found that the prevalence of MetS identified by IDF definition among 215 overweight/obese Mexican children aged 6-12 years was 6.7% [28]. The prevalence of MetS among our overweight and obese participants was 3.5% and 2.1% among children aged 10-11 years and less than 10 years, respectively, which was quite near the prevalence of MetS in overweight and obese children reported in the French study [26] and Mexican study [28].

Applying the IDF criteria for the children equal to or above 10 years old may result in underestimate of the real situation; however, the results at least indicated the early onset of MetS in Chinese children. When applying other definitions proposed by Cook et al., de Ferranti et al. and Ohzeki et al., the estimated prevalence of MetS was much higher. With the increasing of childhood obesity in China and the onset of childhood MetS, the prevalence of the MetS and type 2 diabetes may increase rapidly in China. Morrison JA et al. found that for children with MetS, 68.5% would develop adult MetS and 15.6% would develop type 2 diabetes mellitus in 25-30 years later, while the proportion is 24% and 5%, respectively, for children without MetS developing adult MetS and type 2 diabetes mellitus [31]. The different prevalence of MetS we found may be due to the different prevalence of obesity, of course, we could not rule out the reason of different methodology, for example, enzymatic method was used for HDL-C in our study, but the phosphotungstic acid precipitation method was used for Canadian children. Our results imply that it is urgent for developing countries, especially those undergoing nutritional transition, such as China, to take effective strategies and actions to control and prevent the prevalence of obesity and obesity-related chronic diseases on the onset of the epidemic of obesity.

The age difference in the prevalence of the MetS was found in our study, which is consistent with previous studies. A study of children and adolescents from Iran and Germany found that both Iranian and German younger children aged 6-10 years had lower risk of MetS than their older counterparts aged 10-16 years (1.0% vs. 2.0% for Iranians, 0.1% vs. 0.5% for Germans) [25]. No association of the prevalence of MetS with mother's education and family's income was found in the current study, which was consistent with another study conducted in a national representative sample of Chinese adolescents [12]. As known, many factors contribute to the development of MetS and other chronic disease, such as physical activity level, dietary intake, obesity and social-economic status. Some factors relate with each other and this correlation maybe result in no significant association of risk factors with MetS. This may result in the no-significant relationship between MetS and educational/income level of the parents.

The main strength of the current study was the WC cut-off points for defining abdominal obesity which were developed specifically for Chinese children with the national representative samples in 2010 [21]. Chinese children tend to have lower height and WC at the same age compared with Caucasians due to the ethnic difference in body shape and body composition. Therefore, the definition of central obesity proposed by IDF uses the 90th percentile of WC, rather than cut-off points of WC. The cut-off points of WC employed in our study were developed from a national representative sample (160,225 children and adolescents aged 7-18 years old) and published in 2010. Compared with the other studies which used the cut-off points of WC developed from Caucasian children or non-representative Chinese children to define the MetS among Chinese children, our results seem more valid. There are some limitations in the current study. Firstly, as indicated by the IDF definition, MetS can not be diagnosed for children younger than 10 years old except among children with dyslipidemia, cardiovascular disease, hypertension or obesity. So the interpretation of present results among younger children should be cautiously. The MetS can not be applied to young children. Identifying the cluster of metabolic abnormalities in children may be important for the prevention of chronic diseases in later life, the earlier the better. Secondly, the prevalence of hypertension may be overestimated, because the diagnosis of hypertension was based on the measurements of blood pressure on one occasion, rather than on three different occasions. Finally, the IDF criteria may underestimate the prevalence of MetS because the criteria for hypertension were based on adult cutoff points rather than pediatric specific norms [32], which were supported when applying different criterion for the definition of the MetS. We presented the prevalence of MetS based on different criterion in order to be comparable with other studies. However, it should be cautious of the methodology differences between studies. Another limitation is that our study represents a group of Chinese children with a relatively high socioeconomic status and findings of this analysis might not be generalizable to other populations.

## Conclusions

Based on the large population study, we observed the early onset of MetS in Chinese children. The prevalence of MetS and its five individual abnormalities were all significantly higher among overweight and obese children than that among normal weight children. The proportions of children with at least one or two metabolic abnormalities were relatively high. It is urgent for China to take effective strategies and actions to control and prevent childhood obesity and obesity-related chronic disease in children.

## Competing interests

The authors declare that they have no competing interests.

## Authors' contributions

HX and YL^Aff1 ^carried out statistical analysis and drafted the manuscript. AL, QZ, XH and HF contributed to study design and organization, interpretation of the data and revising the manuscript. DL, JM, GX, YL^Aff4^, HG and TL were the chief investigators at the center level and responsible for the data collection and input. They also contributed to revising the manuscript. GM was the principal investigator of the whole project and responsible for the project design, organization and implementation. He also contributed to interpretation of the data and revising the manuscript. All authors read and approved the final manuscript.

## Pre-publication history

The pre-publication history for this paper can be accessed here:

http://www.biomedcentral.com/1471-2458/12/13/prepub

## Supplementary Material

Additional file 1** Table S1** The Prevalence of the Metabolic Syndrome among children by different definition. Click here for file
