# Supplementary material for: Prevalence of the metabolic syndrome among children from six cities of China
Source: BMC Public Health. 2012 Jan 6;12:13. doi: 10.1186/1471-2458-12-13 (PMC3316129; doi:10.1186/1471-2458-12-13)
Supplement: Additional file 1 Table S1 — The Prevalence of the Metabolic Syndrome among children by different definition. [file 1471-2458-12-13-S1.DOC]

Table S1. The Prevalence of the Metabolic Syndrome among children by different definition

|  | | ≥10 | | | | |  | | <10 | | | | | |
| --- | --- | --- | --- | --- | --- | --- | --- | --- | --- | --- | --- | --- | --- | --- |
|  | N | | IDF | Cook  et al. | Ohzeki  et al. | de Ferranti  et al. | |  | | N | IDF | Cook  et al. | Ohzeki  et al. | de Ferranti  et al. |
| Total | 2647 | | 0.8 | 2.5 | 2.6 | 11.6 | |  | | 6117 | 0.5 | 1.5 | 1.0 | 7.8 |
| ^a^Sex |  | |  |  |  |  | |  | |  |  |  |  |  |
| Boy | 1394 | | 1.0 | 3.3 | 3.2 | 12.2 | |  | | 3101 | 0.5 | 1.7 | 1.2 | 7.6 |
| Girl | 1253 | | 0.6 | 1.6 | 1.8 | 10.9 | |  | | 3016 | 0.4 | 1.2 | 0.8 | 8.0 |
| ^ab^BMI status |  | |  |  |  |  | |  | |  |  |  |  |  |
| Normal weight | 2044 | | 0.05^**^ | 0.4 | 0.6 | 4.4 | |  | | 4813 | 0.04^**^ | 0.4 | 0.4 | 2.8 |
| Overweight | 330 | | 0.9 | 3.0 | 3.0 | 25.8 | |  | | 675 | 0.4 | 0.5 | 0.7 | 18.2 |
| Obesity | 273 | | 6.6 | 17.2 | 16.9 | 48.0 | |  | | 629 | 4.0 | 10.8 | 5.9 | 34.8 |
| ^ab^Mother’s educational level |  | |  |  |  |  | |  | |  |  |  |  |  |
| Low (illiterate) | 28 | | 3.6 | 3.6 | 7.1 | 25.0 | |  | | 52 | 0 | 1.9 | 1.9 | 5.8 |
| Middle (Primary or junior middle school) | 1138 | | 0.8 | 2.1 | 2.4 | 11.7 | |  | | 1854 | 0.8 | 1.4 | 1.0 | 7.6 |
| High (Senior middle school or above) | 1111 | | 1.0 | 2.4 | 2.5 | 10.8 | |  | | 2276 | 0.5 | 1.8 | 1.3 | 8.8 |
| ^ab^Family’s economic level |  | |  |  |  |  | |  | |  |  |  |  |  |
| (Yuan/month/per family member) |  | |  |  |  |  | |  | |  |  |  |  |  |
| ≤1500 | 1042 | | 0.9 | 1.9 | 2.0 | 10.6 | |  | | 1808 | 0.5 | 1.6 | 0.9 | 8.8 |
| 1501-2500 | 617 | | 1.3 | 3.1 | 3.6 | 13.0 | |  | | 1145 | 0.7 | 1.8 | 1.5 | 7.3 |
| >2500 | 610 | | 0.5 | 2.5 | 2.6 | 11.3 | |  | | 1171 | 0.7 | 1.4 | 1.2 | 8.0 |

^*^*p* < 0.05, ^**^*p* < 0.01, compared using generalized linear mixed model.

^a^Adjusted for age and puberty for comparison of different groups.

^b^Adjusted for sex and puberty for comparison of different groups.

^ab^Adjusted for age, sex and puberty for comparison of different groups.
